# All Mold Is Not Alike: The Importance of Intraspecific Diversity in Necrotrophic Plant Pathogens

**DOI:** 10.1371/journal.ppat.1000759

**Published:** 2010-03-26

**Authors:** Heather C. Rowe, Daniel J. Kliebenstein

**Affiliations:** Department of Plant Sciences, University of California, Davis, Davis, California, United States of America; The Fox Chase Cancer Center, United States of America

Pathogens commonly possess naturally occurring intraspecific variation for traits associated with pathogenicity or virulence. Studies of host–pathogen interactions frequently fail to acknowledge this variation, particularly in studies of necrotrophic plant pathogens, where the molecular bases of defense are largely unknown. Necrotrophic plant pathogens, in contrast to obligate parasites of living plant cells known as biotrophs, kill plant cells before consuming them and may survive in the absence of living host cells in dormant or saprophytic states [1]–[4]. Necrotrophs may kill host cells using an array of toxins, although it is also proposed that these pathogens may activate plant immune responses designed to work against biotrophic pathogens, thus encouraging plant cells to kill themselves [5]–[9]. While many pathogen species cannot be clearly classified as either biotrophic or necrotrophic, as they shift lifestyles over the course of interactions with their hosts, commonly recognized necrotrophic plant pathogens include various species of *Botrytis* and *Alternaria*, as well as *Sclerotinia sclerotiorum*, *Pythium irregulare*, and *Plectosphaerella cucurmerina* [2],[10]. Of these, *Botrytis cinerea*, a highly generalist pathogen, and *Alternaria brassicicola*, a specialist pathogen of *Brassica*, dominate research on molecular mechanisms of plant defense against necrotrophic pathogens.

Plant immune responses against biotrophic pathogens are predominantly mediated by specific recognition of the products of pathogen “avirulence” (avr) genes directly or indirectly by the products of plant “resistance” (R) genes; localized cell death is believed to restrict the growth of obligate (biotrophic) parasites [11],[12]. Intraspecific variation in pathogen avr genes is common, as these genes are believed to confer a selective pathogen advantage in the absence of the corresponding plant R gene [13]–[15]. Currently, specific recognition of necrotrophic pathogens by similar mechanisms has not been documented, although similar evolutionary dynamics may shape the interplay between variable plant sensitivity to some necrotroph-produced toxins (called “host selective toxins”) and variable production of these toxins by the pathogen [15]–[17]. This lack of identified specific recognition has generated a prevailing view in the plant molecular defense research community that as necrotrophic pathogens are not reported to engage in specific interactions with host plants, all isolates of a particular necrotrophic pathogen species are equivalent. This opinion manifests itself in a lack of use of necrotrophic diversity in published studies, as well as a lack of reporting of identifying pathogen data, despite published evidence that necrotrophic pathogens show intraspecific variation affecting pathogenesis- or virulence-related traits [18]–[24]. We suggest that the limited use of pathogen diversity biases our understanding of plant–necrotroph interactions. The research community should enforce detailed reporting of identifying pathogen data for studies of plant–necrotroph interactions and encourage the use of multiple pathogen genotypes.

## Lack of Diversity

The majority of studies investigating the molecular bases of plant–necrotroph interactions do not include pathogen variation. Based on a survey of published literature from the last 10 years, fewer than 12% of surveyed studies of plant defense against *Botrytis cinerea*, the most intensively researched plant necrotrophic pathogen as reflected by publication frequency, report experimental results for more than one pathogen isolate (see Text S1). The diversity of *A. brassicicola* represented in the current literature is much lower, as none of the surveyed studies reported data from multiple pathogen isolates and almost half of these studies used the same isolate, MUCL20297. While selection of a particular pathogen isolate as a model or laboratory standard may facilitate comparison among studies performed in different laboratories, data from single isolates are too often represented as informative for the whole pathogen species. If the reference isolate is atypical, misleading conclusions may be drawn regarding the biology of the plant–host interaction, and promising lines of research may be abandoned.

## A Cautionary Example: Resveratrol

The controversial role of phytoalexin defense compounds in providing actual plant defense against pathogens illustrates the importance of including necrotroph variation in studies of plant defense. One phytoalexin compound implicated in plant defense is resveratrol, a stilbenoid phytoalexin produced by *Vitis vinifera* in response to pathogen attack [25],[26]. As the chemical precursors for resveratrol are produced by all plants, transgenic introduction of *V. vinifera* stilbene synthases into several crop plants provided the capacity for heterologous production of this antimicrobial compound [27]. Independent studies of transgenic tomato, barley, and tobacco evaluated resveratrol's efficacy against *B. cinerea*. Intriguingly, the capacity to produce resveratrol enhanced plant resistance to *B. cinerea* in barley and tobacco, but had no significant effect on tomato resistance to *B. cinerea*, despite plant accumulation of resveratrol at concentrations sufficient to inhibit *B. cinerea* growth [28].

While inhibition of *B. cinerea* growth by resveratrol might depend on the host in which it is encountered by the pathogen, the reported capacity of *B. cinerea* to degrade stilbenoid phytoalexins by the action of laccases suggests an alternative explanation [28],[29]. Eight surveyed *B. cinerea* isolates varied in their capacity to degrade resveratrol; this variation was linked to virulence on grape leaves [29]. The studies of resveratrol-producing tobacco and barley do not provide any information about the *B. cinerea* isolate(s) used, and the tomato study reports use of “a spore suspension of field isolates”, possibly representing a mixture of pathogen genotypes [30]–[32]. The observed lack of increased *B. cinerea* resistance in resveratrol-producing tomato plants might result from the presence of resveratrol-degrading *B. cinerea* isolates, while tests of transgenic tobacco and barley used isolates with reduced or no capacity to degrade resveratrol. Without documentation and archiving of *B. cinerea* isolates used, it is impossible to retroactively distinguish whether these conflicting results reflect pathogen isolate differences or differences in plant biochemistry and physiology.

## Lack of Reporting

A lack of reported information about necrotrophic pathogen isolates is a less common, but more troubling, deficiency in the published literature. Approximately 15%–20% of surveyed publications reporting original research on plant defense against either *B. cinerea* or *A. brassicicola* did not provide any description of the pathogen isolate used. Minimally, an isolate name and explicit details of the isolate's source should be provided. In addition, references to source materials or isolation methods should include documentation of how the species identity was confirmed, as pathogens may be difficult to distinguish by morphology or collection host. Additional information, such as collection date, host, and geography, may add valuable context for other researchers, especially for species such as *B. cinerea* where cryptic speciation related to host use and geography have been proposed [33]–[36].

## Steps Forward

Pathogen diversity presents serious challenges and opportunities for understanding pathogen interactions with host defenses. Conclusions drawn from studies employing single, or even multiple, isolates may not accurately represent the biology of the species as a whole. Variation in either the host or the pathogen can alter these relationships and this should be at least acknowledged in biological studies. Further, the lax acknowledgement of genotypic diversity within necrotrophic plant pathogens hinders comparison among studies through both a lack of overlap among experimental isolates used by different research groups and a lack of explicit description of the isolates used. Use of a standardized panel of pathogen isolates is impracticable given restrictions on the import and movement of plant pathogens, and might provide a false resolution to this issue, as the rate of genomic change in these pathogens, particularly in response to selection for laboratory growth, is unknown.

A promising strategy would embrace pathogen diversity to provide a more detailed picture of how plant and necrotrophic pathogen species interact, creating a valuable link between molecular- and population-level studies. This would require preliminary evaluation of diversity in a collection of isolates for a given study trait, followed by detailed characterization of a subset of isolates covering the identified range of trait variation. The paucity of studies employing this strategy likely reflects the effort required to obtain large pathogen collections and the increase in experimental resources required. Minimally, the scientific community and particularly scientific journals should require a detailed description of isolates, including isolate verification and proper referencing, as a prerequisite for publication. Cooperation among laboratories to independently confirm experimental findings should also be encouraged, as this will improve interpretation of single-isolate studies and minimize disagreements caused by pathogen variation.

## Supporting Information

Text S1 Literature reviewed. List of publications reporting original data on plant interactions with necrotrophic fungal pathogens retrieved from ISI Web of Science using the combined topic search terms “Botrytis cinerea”and “plant defense”or “Alternaria brassicicola” and “plant defense”.
(0.08 MB DOC)

## References

[1] Glazebrook J (2005). Contrasting mechanisms of defense against biotrophic and necrotrophic pathogens.. Annu Rev Phytopathol.

[2] Oliver RP, Ipcho SVS (2004). Arabidopsis pathology breathes new life into the necrotrophs-vs.-biotrophs classification of fungal pathogens.. Mol Plant Pathol.

[3] van Kan JAL (2006). Licensed to kill: the lifestyle of a necrotrophic plant pathogen.. Trends Plant Sci.

[4] Williamson B, Tudzynsk B, Tudzynski P, van Kan JAL (2007). *Botrytis cinerea*: the cause of grey mould disease.. Mol Plant Pathol.

[5] Dickman MB, Park YK, Oltersdorf T, Li W, Clemente T (2001). Abrogation of disease development in plants expressing animal antiapoptotic genes.. Proc Natl Acad Sci USA.

[6] Govrin EM, Levine A (2000). The hypersensitive response facilitates plant infection by the necrotrophic pathogen *Botrytis cinerea*.. Curr Biol.

[7] Govrin EM, Rachmilevitch S, Tiwari BS, Soloman M, Levine A (2006). An elicitor from *Botrytis cinerea* induces the hypersensitive response in *Arabidopsis thaliana* and other plants and promotes the gray mold disease.. Phytopathology.

[8] Kliebenstein DJ, Rowe HC (2008). Ecological costs of biotrophic versus necrotrophic pathogen resistance, the hypersensitive response and signal transduction.. Plant Science.

[9] Wang WM, Devoto A, Turner JG, Xiao SY (2007). Expression of the membrane-associated resistance protein RPW8 enhances basal defense against biotrophic pathogens.. Mol Plant-Microbe Interact.

[10] Thaler JS, Owen B, Higgins VJ (2004). The role of the jasmonate response in plant susceptibility to diverse pathogens with a range of lifestyles.. Plant Physiol.

[11] Dangl JL, Jones JDG (2001). Plant pathogens and integrated defence responses to infection.. Nature.

[12] Jones JDG, Dangl JL (2006). The plant immune system.. Nature.

[13] Kamoun S (2007). Groovy times: filamentous pathogen effectors revealed.. Curr Opin Plant Biol.

[14] Salvaudon L, Giraud T, Shykoff JA (2008). Genetic diversity in natural populations: a fundamental component of plant-microbe interactions.. Curr Opin Plant Biol.

[15] Stukenbrock EH, McDonald BA (2009). Population Genetics of Fungal and Oomycete Effectors Involved in Gene-for-Gene Interactions.. MolPlant-Microbe Interact.

[16] Friesen TL, Faris JD, Solomon PS, Oliver RP (2008). Host-specific toxins: effectors of necrotrophic pathogenicity.. Cellu Microbiol.

[17] Lawrence CB, Mitchell TK, Craven KD, Cho Y, Cramer RA (2008). At death's door: Alternaria pathogenicity mechanisms.. Plant Pathol J.

[18] Derckel JP, Baillieul F, Manteau S, Audran JC, Haye B (1999). Differential induction of grapevine defenses by two strains of *Botrytis cinerea*.. Phytopathology.

[19] Kliebenstein DJ, Rowe HC, Denby KJ (2005). Secondary metabolites influence Arabidopsis/Botrytis interactions: variation in host production and pathogen sensitivity.. Plant J.

[20] Quidde T, Osbourn AE, Tudzynski P (1998). Detoxification of alpha-tomatine by *Botrytis cinerea*.. Physiol Mol Plant Pathol.

[21] Schoonbeek H, Del Sorbo G, De Waard MA (2001). The ABC transporter *BcatrB* affects the sensitivity of *Botrytis cinerea* to the phytoalexin resveratrol and the fungicide fenpiclonil.. Mol Plant-Microbe Interact.

[22] Sellam A, Iacomi-Vasilescu B, Hudhomme P, Simoneau P (2007). In vitro antifungal activity of brassinin, camalexin and two isothiocyanates against the crucifer pathogens *Alternaria brassicicola* and *Alternaria brassicae*.. Plant Pathol.

[23] Siewers V, Viaud M, Jimenez-Teja D, Collado IG, Gronover CS (2005). Functional analysis of the cytochrome P450 monooxygenase gene *bcbot1* of *Botrytis cinerea* indicates that botrydial is a strain-specific virulence factor.. Mol Plant-Microbe Interact.

[24] Unger C, Kleta S, Jandl G, von Tiedemann A (2005). Suppression of the defence-related oxidative burst in bean leaf tissue and bean suspension cells by the necrotrophic pathogen *Botrytis cinerea*.. J Phytopathol.

[25] Jeandet P, Bessis R, Sbaghi M, Meunier P (1995). Production of the phytoalexin resveratrol by grapes as a response to Botrytis attack under natural conditions.. J Phytopathol.

[26] Kopp P (1998). Resveratrol, a phytoestrogen found in red wine. A possible explanation for the conundrum of the ‘French paradox’?. Eur J Endocrinol.

[27] Essenberg M (2001). Prospects for strengthening plant defenses through phytoalexin engineering.. Physiol Mol Plant Pathol.

[28] Adrian M, Rajaei H, Jeandet P, Veneau J, Bessis R (1998). Resveratrol oxidation in *Botrytis cinerea* conidia.. Phytopathology.

[29] Sbaghi M, Jeandet P, Bessis R, Leroux P (1996). Degradation of stilbene-type phytoalexins in relation to the pathogenicity of *Botrytis cinerea* to grapevines.. Plant Pathol.

[30] Thomzik JE, Stenzel K, Stocker R, Schreier PH, Hain R (1997). Synthesis of a grapevine phytoalexin in transgenic tomatoes (*Lycopersicon esculentum* Mill.) conditions resistance against *Phytophthora infestans*.. Physiol Mol Plant Pathol.

[31] Hain R, Reif HJ, Krause E, Langebartels R, Kindl H (1993). Disease resistance results from foreign phytoalexin expression in a novel plant.. Nature.

[32] Leckband G, Lörz H (1998). Transformation and expression of a stilbene synthase gene of *Vitis vinifera* L. in barley and wheat for increased fungal resistance.. Theor Appl Genet.

[33] Albertini C, Thebaud G, Fournier E, Leroux P (2002). Eburicol 14α-demethylase gene (*CYP51*) polymorphism and speciation in *Botrytis cinerea*.. Mycol Res.

[34] Cettul E, Rekab D, Locci R, Firrao R (2008). Evolutionary analysis of endopolygalacturonase-encoding genes of *Botrytis cinerea*.. Mol Plant Pathol.

[35] Fournier E, Giraud T, Albertini C, Brygoo Y (2005). Partition of the *Botrytis cinerea* complex in France using multiple gene genealogies.. Mycologia.

[36] Giraud T, Fortini D, Levis C, Lamarque C, Leroux P (1999). Two sibling species of the *Botrytis cinerea* complex, *transposa* and *vacuma*, are found in sympatry on numerous host plants.. Phytopathology.

