# Supplementary material for: All Mold Is Not Alike: The Importance of Intraspecific Diversity in Necrotrophic Plant Pathogens
Source: PLoS Pathog. 2010 Mar 26;6(3):e1000759. doi: 10.1371/journal.ppat.1000759 (PMC2845657; doi:10.1371/journal.ppat.1000759)
Supplement: Text S1 — Literature reviewed. List of publications reporting original data on plant interactions with necrotrophic fungal pathogens retrieved from ISI Web of Science using the combined topic search terms “Botrytis cinerea”and “plant defense”or “Alternaria brassicicola” and “plant defense”. (0.08 MB DOC) [file ppat.1000759.s001.doc]

**SUPPLEMENTAL TEXT**

ISI's Web of Knowledge Database (apps.isiknowledge.com) was queried using the combined search terms “Botrytis cinerea” and “plant defense”. The publications recovered using these search terms were manually screened to eliminate publications that did not report original data on *B. cinerea*—plant interaction. Identifying pathogen information was then extracted from 130 publications (listed below), from the following data classes: isolate name (reported in 67% of publications), host (23%), isolate source (44%), references to previously published experiments using an isolate (24%), and geographic location of collection (6%). Only 15 publications reported experiments using two or more isolates. Twenty-three studies (17.7%) provided no information about the *B. cinerea* isolate(s) used.

A similar approach was used to query the potential influence of genetic variation in *A. brassicicola* on our interpretation of plant mechanistic studies. Querying ISI's Web of Knowledge Database using the combined search terms “Alternaria brassicicola” and “plant defense” recovered only 40 publications (listed below) after removal of publications not reporting original data relevant to *A. brassicicola—*plant interactions. Identifying pathogen data included: isolate name (reported in 70% of publications), host (5%), isolate source (45%), and references to previously published experiments using the isolate (33%). No studies reported data from multiple isolates. Six studies (15%) did not provide sufficient data to identify or infer the pathogen isolate(s) used. Expanding the search by using only the search term “Alternaria brassicicola” yielded 169 publications; two of these publications report data from experiments using multiple *A. brassicicola* isolates.

**Publications reporting data on plant—*B. cinerea* interaction surveyed for isolate information:**

1. AbuQamar S, Chen X, Dhawan R, Bluhm B, Salmeron J, et al. (2006) Expression profiling and mutant analysis reveals complex regulatory networks involved in Arabidopsis response to Botrytis infection. Plant J 48: 28-44.

2. Aerts AM, Thevissen K, Bresseleers SM, Sels J, Wouters P, et al. (2007) *Arabidopsis thaliana* plants expressing human beta-defensin-2 are more resistant to fungal attack: functional homology between plant and human defensins. Plant Cell Rep 26: 1391-1398.

3. Aguero CB, Uratsu SL, Greve C, Powell ALT, Labavitch JM, et al. (2005) Evaluation of tolerance to Pierce's disease and Botrytis in transgenic plants of *Vitis vinifera* L. expressing the pear PGIP gene. Mol Plant Pathol 6: 43-51.

4. Asselbergh B, Curvers K, Franca SC, Audenaert K, Vuylsteke M, et al. (2007) Resistance to *Botrytis cinerea* in *sitiens*, an abscisic acid-deficient tomato mutant, involves timely production of hydrogen peroxide and cell wall modifications in the epidermis. Plant Physiol 144: 1863-1877.

5. Audenaert K, De Meyer GB, Hofte MM (2002) Abscisic acid determines basal susceptibility of tomato to *Botrytis cinerea* and suppresses salicylic acid-dependent signaling mechanisms. Plant Physiol 128: 491-501.

6. Audenaert K, Pattery T, Cornelis P, Hofte M (2002) Induction of systemic resistance to *Botrytis cinerea* in tomato by *Pseudomonas aeruginosa* 7NSK2: Role of salicylic acid, pyochelin, and pyocyanin. Mol Plant-Microbe Interact 15: 1147-1156.

7. Azevedo H, Conde C, Geros H, Tavares RM (2006) The non-host pathogen *Botrytis cinerea* enhances glucose transport in *Pinus pinaster* suspension-cultured cells. Plant Cell Physiol 47: 290-298.

8. Azevedo H, Lino-Neto T, Tavares RM (2008) The necrotroph *Botrytis cinerea* induces a non-host type II resistance mechanism in *Pinus pinaster* suspension-cultured cells. Plant Cell Physiol 49: 386-395.

9. Aziz A, Gauthier A, Bezler A, Poinssot B, Joubert JM, et al. (2007) Elicitor and resistance-inducing activities of beta-1,4 cellodextrins in grapevine, comparison with beta-1,3 glucans and alpha-1,4 oligogalacturonides. J Exper Bot 58: 1463-1472.

10. Aziz A, Poinssot B, Daire X, Adrian M, Bezier A, et al. (2003) Laminarin elicits defense responses in grapevine and induces protection against *Botrytis cinerea* and *Plasmopara viticola*. Mol Plant-Microbe Interact 16: 1118-1128.

11. Aziz A, Trotel-Aziz P, Dhuicq L, Jeandet P, Couderchet M, et al. (2006) Chitosan oligomers and copper sulfate induce grapevine defense reactions and resistance to gray mold and downy mildew. Phytopathology 96: 1188-1194.

12. Barto EK, Cipollini D (2005) Testing the optimal defense theory and the growth-differentiation balance hypothesis in *Arabidopsis thaliana*. Oecologia 146: 169-178.

13. Berrocal-Lobo M, Molina A, Solano R (2002) Constitutive expression of ETHYLENE-RESPONSE-FACTOR1 in Arabidopsis confers resistance to several necrotrophic fungi. Plant J 29: 23-32.

14. Bessire M, Chassot C, Jacquat AC, Humphry M, Borel S, et al. (2007) A permeable cuticle in Arabidopsis leads to a strong resistance to *Botrytis cinerea*. EMBO J 26: 2158-2168.

15. Brouwer M, Lievens B, Van Hemelrijck W, Van den Ackerveken G, Cammue BPA, et al. (2003) Quantification of disease progression of several microbial pathogens on *Arabidopsis thaliana* using real-time fluorescence PCR. FEMS Microbiol Lett 228: 241-248.

16. Brutus A, Reca IB, Herga S, Mattei B, Puigserver A, et al. (2005) A family 11 xylanase from the pathogen *Botrytis cinerea* is inhibited by plant endoxylanase inhibitors XIP-I and TAXI-I. Biochem Biophys Res Comm 337: 160-166.

17. Calo L, Garcia I, Gotor C, Romero LC (2006) Leaf hairs influence phytopathogenic fungus infection and confer an increased resistance when expressing a Trichoderma alpha-1,3-glucanase. J Exper Bot 57: 3911-3920.

18. Caruso C, Caporale C, Chilosi G, Vacca F, Bertini L, et al. (1996) Structural and antifungal properties of a pathogenesis-related protein from wheat kernel. J Prot Chem 15: 35-44.

19. Chague V, Danit LV, Siewers V, Gronover CS, Tudzynski P, et al. (2006) Ethylene sensing and gene activation in *Botrytis cinerea*: A missing link in ethylene regulation of fungus-plant interactions? Mol Plant-Microbe Interact 19: 33-42.

20. Chen H, Xiao X, Wang J, Wu LJ, Zheng ZM, et al. (2008) Antagonistic effects of volatiles generated by *Bacillus subtilis* on spore germination and hyphal growth of the plant pathogen, *Botrytis cinerea*. Biotechnol Lett 30: 919-923.

21. Coego A, Ramirez V, Gil MJ, Flors V, Mauch-Mani B, et al. (2005) An Arabidopsis homeodomain transcription factor, OVEREXPRESSOR OFCATIONIC PEROXIDASE 3, mediates resistance to infection by necrotrophic pathogens. Plant Cell 17: 2123-2137.

22. Company P, Gonzalez-Bosch C (2003) Identification of a copper chaperone from tomato fruits infected with *Botrytis cinerea* by differential display. Biochem Biophys Res Comm 304: 825-830.

23. Corrado G, Bovi PD, Ciliento R, Gaudio L, Di Maro A, et al. (2005) Inducible expression of a *Phytolacca heterotepala* ribosome-inactivating protein leads to enhanced resistance against major fungal pathogens in tobacco. Phytopathology 95: 206-215.

24. Daulagala P, Allan EJ (2003) L-form bacteria of *Pseudomonas syringae* pv. phaseolicola induce chitinases and enhance resistance to *Botrytis cinerea* infection in Chinese cabbage. Physiol Mol Plant Pathol 62: 253-263.

25. de Leon IP, Oliver JP, Castro A, Gaggero C, Bentancor M, et al. (2007) *Erwinia carotovora* elicitors and *Botrytis cinerea* activate defense responses in *Physcomitrella patens*. BMC Plant Biol 7: 52.

26. Deak M, Horvath GV, Davletova S, Torok K, Sass L, et al. (1999) Plants ectopically expressing the iron-binding protein, ferritin, are tolerant to oxidative damage and pathogens. Nature Biotechnol 17: 192-196.

27. Deighton N, Muckenschnabel I, Goodman BA, Williamson B (1999) Lipid peroxidation and the oxidative burst associated with infection of *Capsicum annuum* by *Botrytis cinerea*. Plant J 20: 485-492.

28. Derckel JP, Baillieul F, Manteau S, Audran JC, Haye B, et al. (1999) Differential induction of grapevine defenses by two strains of *Botrytis cinerea*. Phytopathology 89: 197-203.

29. Diaz J, ten Have A, van Kan JAL (2002) The role of ethylene and wound signaling in resistance of tomato to *Botrytis cinerea*. Plant Physiol 129: 1341-1351.

30. El Oirdi M, Bouarab K (2007) Plant signalling components EDS1 and SGT1 enhance disease caused by the necrotrophic pathogen *Botrytis cinerea*. New Phytol 175: 131-139.

31. Ferrari S, Galletti R, Denoux C, De Lorenzo G, Ausubel FM, et al. (2007) Resistance to *Botrytis cinerea* induced in Arabidopsis by elicitors is independent of salicylic acid, ethylene, or jasmonate signaling but requires PHYTOALEXIN DEFICIENT3. Plant Physiol 144: 367-379.

32. Ferrari S, Galletti R, Pontiggia D, Manfredini C, Lionetti V, et al. (2008) Transgenic expression of a fungal endo-polygalacturonase increases plant resistance to pathogens and reduces auxin sensitivity. Plant Physiol 146: 669-681.

33. Ferrari S, Galletti R, Vairo D, Cervone F, De Lorenzo G (2006) Antisense expression of the *Arabidopsis thaliana* AtPGIP1 gene reduces polygalacturonase-inhibiting protein accumulation and enhances susceptibility to *Botrytis cinerea*. Mol Plant-Microbe Interact 19: 931-936.

34. Ferrari S, Plotnikova JM, De Lorenzo G, Ausubel FM (2003) Arabidopsis local resistance to *Botrytis cinerea* involves salicylic acid and camalexin and requires *EDS4* and *PAD2* but not *SID2, EDS5*, or *PAD4*. Plant J 35: 193-205.

35. Ferrari S, Vairo D, Ausubel FM, Cervone F, De Lorenzo G (2003) Tandemly duplicated arabidopsis genes that encode polygalacturonase-inhibiting proteins are regulated coordinately by different signal transduction pathways in response to fungal infection. Plant Cell 15: 93-106.

36. Flors V, Leyva MD, Vicedo B, Finiti I, Real MD, et al. (2007) Absence of the endo-beta-1,4-glucanases Cel1 and Cel2 reduces susceptibility to *Botrytis cinerea* in tomato. Plant J 52: 1027-1040.

37. Francia D, Demaria D, Calderini O, Ferraris L, Valentino D, et al. (2007) Wounding induces resistance to pathogens with different lifestyles in tomato: role of ethylene in cross-protection. Plant Cell Environ 30: 1357-1365.

38. Girault T, Francois J, Rogniaux H, Pascal S, Delrot S, et al. (2008) Exogenous application of a lipid transfer protein-jasmonic acid complex induces protection of grapevine towards infection by *Botrytis cinerea*. Plant Physiol Biochem 46: 140-149.

39. Gomes E, Sagot E, Gaillard C, Laquitaine L, Poinssot B, et al. (2003) Nonspecific lipid-transfer protein genes expression in grape (Vitis sp.) cells in response to fungal elicitor treatments. Mol Plant-Microbe Interact 16: 456-464.

40. Gonzalez J, Reyes F, Salas C, Santiago M, Codriansky Y, et al. (2006) *Arabidopsis thaliana*: A model host plant to study plant-pathogen interaction using Chilean field isolates of *Botrytis cinerea*. Biol Res 39: 221-228.

41. Govrin EM, Levine A (2000) The hypersensitive response facilitates plant infection by the necrotrophic pathogen *Botrytis cinerea*. Curr Biol 10: 751-757.

42. Govrin EM, Levine A (2002) Infection of *Arabidopsis* with a necrotrophic pathogen, *Botrytis cinerea*,elicits various defense responses but does not induce systemic acquired resistance (SAR). Plant Mol Biol 48: 267–276.

43. Govrin EM, Rachmilevitch S, Tiwari BS, Soloman M, Levine A (2006) An elicitor from *Botrytis cinerea* induces the hypersensitive response in *Arabidopsis thaliana* and other plants and promotes the gray mold disease. Phytopathology 96: 299-307.

44. Grant JJ, Chini A, Basu D, Loake GJ (2003) Targeted activation tagging of the Arabidopsis NBS-LRR gene, ADR1, conveys resistance to virulent pathogens. Mol Plant-Microbe Interact 16: 669-680.

45. Gronover CS, Kasulke D, Tudzynski P, Tudzynski B (2001) The role of G protein alpha subunits in the infection process of the gray mold fungus *Botrytis cinerea*. Mol Plant-Microbe Interact 14: 1293-1302.

46. Gronover CS, Schorn C, Tudzynski B (2004) Identification of *Botrytis cinerea* genes up-regulated during infection and controlled by the Galpha subunit BCG1 using suppression subtractive hybridization (SSH). Mol Plant-Microbe Interact 17: 537-546.

47. Guimaraes RL, Chetelat RT, Stotz HU (2004) Resistance to *Botrytis cinerea* in *Solanum lycopersicoides* is dominant in hybrids with tomato, and involves induced hyphal death. Eur J Plant Pathol 110: 13-23.

48. Hammer PE, Evensen KB (1994) Differences between rose cultivars in susceptibility to infection by *Botrytis cinerea*. Phytopathology 84: 1305-1312.

49. Hashim M, Roberts JA, Rossall S, Dickinson MJ (1997) Leaflet abscission and phytoalexin production during the response of two faba bean breeding lines to Botrytis infection. Plant Pathol 46: 989-996.

50. Hayashi K, Schoonbeek HJ, De Waard MA (2002) Bcmfs1, a novel major facilitator superfamily transporter from *Botrytis cinerea*, provides tolerance towards the natural toxic compounds camptothecin and cercosporin and towards fungicides. Appl Environ Microbiol 68: 4996-5004.

51. He PQ, Tian L, Chen KS, Hao LH, Li GY (2006) Induction of volatile organic compounds of *Lycopersicon esculentum* Mill. and its resistance to *Botrytis cinerea* Pers. by burdock oligosaccharide. J Integr Plant Biol 48: 550-557.

52. Hoffland E, van Beusichem ML, Jeger MJ (1999) Nitrogen availability and susceptibility of tomato leaves to *Botrytis cinerea*. Plant and Soil 210: 263-272.

53. Hou CT, Forman RJ (2000) Growth inhibition of plant pathogenic fungi by hydroxy fatty acids. J Indust Microbiol Biotechnol 24: 275-276.

54. Joubert DA, Slaughter AR, Kemp G, Becker JVW, Krooshof GH, et al. (2006) The grapevine polygalacturonase-inhibiting protein (VvPGIP1) reduces *Botrytis cinerea* susceptibility in transgenic tobacco and differentially inhibits fungal polygalacturonases. Transgen Res 15: 687-702.

55. Kachroo A, Lapchyk L, Fukushige H, Hildebrand D, Klessig D, et al. (2003) Plastidial fatty acid signaling modulates salicylic acid- and jasmonic acid-mediated defense pathways in the Arabidopsis *ssi2* mutant. Plant Cell 15: 2952-2965.

56. Kachroo P, Shanklin J, Shah J, Whittle EJ, Klessig DF (2001) A fatty acid desaturase modulates the activation of defense signaling pathways in plants. Proc Natl Acad Sci USA 98: 9448-9453.

57. Keller H, Pamboukdjian N, Ponchet M, Poupet A, Delon R, et al. (1999) Pathogen-induced elicitin production in transgenic tobacco generates a hypersensitive response and nonspecific disease resistance. Plant Cell 11: 223-235.

58. Kishimoto K, Matsui K, Ozawa R, Takabayashi J (2005) Volatile C6-aldehydes and allo-ocimene activate defense genes and induce resistance against *Botrytis cinerea* in *Arabidopsis thaliana*. Plant Cell Physiol 46: 1093-1102.

59. Kishimoto K, Matsui K, Wawa R, Takabayashi J (2006) Components of C6-aldehyde-induced resistance in *Arabidopsis thaliana* against a necrotrophic fungal pathogen, *Botrytis cinerea*. Plant Sci 170: 715-723.

60. Kliebenstein DJ, Rowe HC, Denby KJ (2005) Secondary metabolites influence Arabidopsis/Botrytis interactions: variation in host production and pathogen sensitivity. Plant J 44: 25-36.

61. Kuzniak E, Sklodowska M (2001) Ascorbate, glutathione and related enzymes in chloroplasts of tomato leaves infected by *Botrytis cinerea*. Plant Sci 160: 723-731.

62. Kuzniak E, Sklodowska M (2004) The effect of *Botrytis cinerea* infection on the antioxidant profile of mitochondria from tomato leaves. J Exper Bot 55: 605-612.

63. Kuzniak E, Sklodowska M (2005) Fungal pathogen-induced changes in the antioxidant systems of leaf peroxisomes from infected tomato plants. Planta 222: 192-200.

64. Laquitaine L, Gomes E, Francois J, Marchive C, Pascal S, et al. (2006) Molecular basis of ergosterol-induced protection of grape against *Botrytis cinerea*: Induction of type I LTP promoter activity, WRKY, and stilbene synthase gene expression. Mol Plant-Microbe Interact 19: 1103-1112.

65. Lee J, Nam J, Park HC, Na G, Miura K, et al. (2007) Salicylic acid-mediated innate immunity in Arabidopsis is regulated by SIZ1 SUMO E3 ligase. Plant J 49: 79-90.

66. Lee SC, Hwang BK (2006) CASAR82A, a pathogen-induced pepper SAR8.2, exhibits an antifungal activity and its overexpression enhances disease resistance and stress tolerance. Plant Mol Biol 61: 95-109.

67. Lehr NA, Schrey SD, Hampp R, Tarkka MT (2008) Root inoculation with a forest soil streptomycete leads to locally and systemically increased resistance against phytopathogens in Norway spruce. New Phytol 177: 965-976.

68. Lionetti V, Raiola A, Camardella L, Giovane A, Obel N, et al. (2007) Overexpression of pectin methylesterase inhibitors in Arabidopsis restricts fungal infection by *Botrytis cinerea*. Plant Physiol 143: 1871-1880.

69. Llorente F, Alonso-Blanco C, Sanchez-Rodriguez C, Jorda L, Molina A (2005) ERECTA receptor-like kinase and heterotrimeric G protein from Arabidopsis are required for resistance to the necrotrophic fungus *Plectosphaerella cucumerina*. Plant J 43: 165-180.

70. Lopez-Garcia B, Gonzalez-Candelas L, Perez-Paya E, Marcos JF (2000) Identification and characterization of a hexapeptide with activity against phytopathogenic fungi that cause postharvest decay in fruits. Mol Plant-Microbe Interact 13: 837-846.

71. Magnin-Robert M, Trotel-Aziz P, Quantinet D, Biagianti S, Aziz A (2007) Biological control of *Botrytis cinerea* by selected grapevine-associated bacteria and stimulation of chitinase and beta-1,3 glucanase activities under field conditions. Eur J Plant Pathol 118: 43-57.

72. Malolepsza U, Urbanek H (2000) The oxidants and antioxidant enzymes in tomato leaves treated with o-hydroxyethylorutin and infected with *Botrytis cinerea*. Eur J Plant Pathol 106: 657-665.

73. Malolepsza U, Urbanek H (2002) o-Hydroxyethylorutin-mediated enhancement of tomato resistance to *Botrytis cinerea* depends on a burst of reactive oxygen species. J Phytopathol 150: 616-624.

74. Manfredini C, Sicilia F, Ferrari S, Pontiggia D, Salvi G, et al. (2005) Polygalacturonase-inhibiting protein 2 of *Phaseolus vulgaris* inhibits BcPG1, a polygalacturonase of *Botrytis cinerea* important for pathogenicity, and protects transgenic plants from infection. Physiol Mol Plant Pathol 67: 108-115.

75. Manners JM, Penninckx I, Vermaere K, Kazan K, Brown RL, et al. (1998) The promoter of the plant defensin gene PDF1.2 from Arabidopsis is systemically activated by fungal pathogens and responds to methyl jasmonate but not to salicylic acid. Plant Mol Biol 38: 1071-1080.

76. Mehli L, Schaart JG, Kjellsen TD, Tran DH, Salentijn EMJ, et al. (2004) A gene encoding a polygalacturonase-inhibiting protein (PGIP) shows developmental regulation and pathogen-induced expression in strawberry. New Phytol 163: 99-110.

77. Meir S, Droby S, Davidson H, Alsevia S, Cohen L, et al. (1998) Suppression of Botrytis rot in cut rose flowers by postharvest application of methyl jasmonate. Postharvest Biol Technol 13: 235-243.

78. Mengiste T, Chen X, Salmeron J, Dietrich R (2003) The BOTRYTIS SUSCEPTIBLE1 gene encodes an R2R3MYB transcription factor protein that is required for biotic and abiotic stress responses in Arabidopsis. Plant Cell 15: 2551-2565.

79. Mohamed N, Lherminier J, Farmer MJ, Fromentin J, Beno N, et al. (2007) Defense responses in grapevine leaves against *Botrytis cinerea* induced by application of a *Pythium oligandrum* strain or its elicitin, oligandrin, to roots. Phytopathology 97: 611-620.

80. Murphy AM, Holcombe LJ, Carr JP (2000) Characteristics of salicylic acid-induced delay in disease caused by a necrotrophic fungal pathogen in tobacco. Physiol Mol Plant Pathol 57: 47-54.

81. Murray SL, Adams N, Kliebenstein DJ, Loake GJ, Denby KJ (2005) A constitutive PR-1::luciferase expression screen identifies Arabidopsis mutants with differential disease resistance to both biotrophic and necrotrophic pathogens. Mol Plant Pathol 6: 31-41.

82. Mzid R, Marchive C, Blancard D, Deluc L, Barrieu F, et al. (2007) Overexpression of VvWRKY2 in tobacco enhances broad resistance to necrotrophic fungal pathogens. Physiol Plant 131: 434-447.

83. Nandi A, Kachroo P, Fukushige H, Hildebrand DF, Klessig DF, et al. (2003) Ethylene and jasmonic acid signaling affect the NPR1-independent expression of defense genes without impacting resistance to *Pseudomonas syringae* and *Peronospora parasitica* in the Arabidopsis *ssi1* mutant. Mol Plant-Microbe Interact 16: 588-599.

84. Nandi A, Krothapaili K, Buseman CM, Li MY, Welti R, et al. (2003) Arabidopsis sfd mutants affect plastidic lipid composition and suppress dwarfing, cell death, and the enhanced disease resistance phenotypes resulting from the deficiency of a fatty acid desaturase. Plant Cell 15: 2383-2398.

85. Nandi A, Moeder W, Kachroo P, Klessig DF, Shah J (2005) Arabidopsis *ssi2*-conferred susceptibility to *Botrytis cinerea* is dependent on EDS5 and PAD4. Mol Plant-Microbe Interact 18: 363-370.

86. Navazio L, Baldan B, Moscatiello R, Zuppini A, Woo SL, et al. (2007) Calcium-mediated perception and defense responses activated in plant cells by metabolite mixtures secreted by the biocontrol fungus *Trichoderma atroviride*. BMC Plant Biol 7.

87. Nickstadt A, Thomma B, Feussner I, Kangasjarvi J, Zeier J, et al. (2004) The jasmonate-insensitive mutant *jin1* shows increased resistance to biotrophic as well as necrotrophic pathogens. Mol Plant Pathol 5: 425-434.

88. Nurmberg PL, Knox KA, Yun BW, Morris PC, Shafiei R, et al. (2007) The developmental selector AS1 is an evolutionarily conserved regulator of the plant immune response. Proc Natl Acad Sci USA 104: 18795-18800.

89. Patykowski J, Urbanek H (2003) Activity of enzymes related to H2O2 generation and metabolism in leaf apoplastic fraction of tomato leaves infected with *Botrytis cinerea*. J Phytopathol 151: 153-161.

90. Perkovskaya GY, Kravchuk ZN, Grodzinsky DM, Dmitriev AP (2004) Induction of reactive oxygen species and phytoalexins in onion (*Allium cepa*) cell culture by biotic elicitors derived from the fungus *Botrytis cinerea*. Russ J Plant Physiol 51: 609-614.

91. Pernas M, Lopez-Solanilla E, Sanchez-Monge R, Salcedo G, Rodriguez-Palenzuela P (1999) Antifungal activity of a plant cystatin. Mol Plant-Microbe Interact 12: 624-627.

92. Petaisto RL, Heiskanen J, Pulkkinen A (2004) Susceptibility of Norway spruce seedlings to grey mould in the greenhouse during the first growing season. Scand J Forest Res 19: 30-37.

93. Poinssot B, Vandelle E, Bentejac M, Adrian M, Levis C, et al. (2003) The endopolygalacturonase 1 from *Botrytis cinerea* activates grapevine defense reactions unrelated to its enzymatic activity. Mol Plant-Microbe Interact 16: 553-564.

94. Powell ALT, van Kan J, ten Have A, Visser J, Greve LC, et al. (2000) Transgenic expression of pear PGIP in tomato limits fungal colonization. Mol Plant-Microbe Interact 13: 942-950.

95. Quidde T, Buttner P, Tudzynski P (1999) Evidence for three different specific saponin-detoxifying activities in *Botrytis cinerea* and cloning and functional analysis of a gene coding for a putative avenacinase. Eur J Plant Pathol 105: 273-283.

96. Quidde T, Osbourn AE, Tudzynski P (1998) Detoxification of alpha-tomatine by *Botrytis cinerea*. Physiol Mol Plant Pathol 52: 151-165.

97. Raacke IC, Mueller MJ, Berger S (2006) Defects in allene oxide synthase and 12-oxa-phytodienoic acid reductase alter the resistance to *Pseudomonas syringae* and *Botrytis cinerea*. J Phytopathol 154: 740-744.

98. Real MD, Company P, Garcia-Agustin P, Bennett AB, Gonzalez-Bosch C (2004) Characterization of tomato endo-beta-1,4-glucanase Cel1 protein in fruit during ripening and after fungal infection. Planta 220: 80-86.

99. Robert N, Roche K, Lebeau Y, Breda C, Boulay M, et al. (2002) Expression of grapevine chitinase genes in berries and leaves infected by fungal or bacterial pathogens. Plant Sci 162: 389-400.

100. Rojo E, Martin R, Carter C, Zouhar J, Pan SQ, et al. (2004) VPE gamma exhibits a caspase-like activity that contributes to defense against pathogens. Curr Biol 14: 1897-1906.

101. Samac DA, Shah DM (1994) Effect of chitinase antisense RNA expression on disease susceptibility of Arabidopsis plants. Plant Mol Biol 25: 587-596.

102. Schoonbeek H, Del Sorbo G, De Waard MA (2001) The ABC transporter *BcatrB* affects the sensitivity of *Botrytis cinerea* to the phytoalexin resveratrol and the fungicide fenpiclonil. Mol Plant-Microbe Interact 14: 562-571.

103. Schoonbeek HJ, Jacquat-Bovet AC, Mascher F, Metraux JP (2007) Oxalate-degrading bacteria can protect *Arabidopsis thaliana* and crop plants against *Botrytis cinerea*. Mol Plant-Microbe Interact 20: 1535-1544.

104. Schouten A, van Baarlen P, van Kan JAL (2008) Phytotoxic Nep1-like proteins from the necrotrophic fungus *Botrytis cinerea* associate with membranes and the nucleus of plant cells. New Phytol 177: 493-505.

105. Schweighofer A, Kazanaviciute V, Scheikl E, Teige M, Doczi R, et al. (2007) The PP2C-type phosphatase AP2C1, which negatively regulates MPK4 and MPK6, modulates innate immunity, jasmonic acid, and ethylene levels in Arabidopsis. Plant Cell 19: 2213-2224.

106. Segura A, Moreno M, Madueno F, Molina A, Garcia-Olmedo F (1999) Snakin-1, a peptide from potato that is active against plant pathogens. Mol Plant-Microbe Interact 12: 16-23.

107. Seo HS, Song JT, Cheong JJ, Lee YH, Lee YW, et al. (2001) Jasmonic acid carboxyl methyltransferase: A key enzyme for jasmonate-regulated plant responses. Proc Natl Acad Sci USA 98: 4788-4793.

108. Sicilia F, Fernandez-Recio J, Caprari C, De Lorenzo G, Tsernoglou D, et al. (2005) The polygalacturonase-inhibiting protein PGIP2 of Phaseolus vulgaris has evolved a mixed mode of inhibition of endopolygalacturonase PG1 of *Botrytis cinerea*. Plant Physiol 139: 1380-1388.

109. Siewers V, Viaud M, Jimenez-Teja D, Collado IG, Gronover CS, et al. (2005) Functional analysis of the cytochrome P450 monooxygenase gene *bcbot1* of *Botrytis cinerea* indicates that botrydial is a strain-specific virulence factor. Mol Plant-Microbe Interact 18: 602-612.

110. Sohn S, Kim Y, Kim B, Lee S, Lim CK, et al. (2007) Transgenic tobacco expressing the hrpN(EP) gene from *Erwinia pyrifoliae* triggers defense responses against *Botrytis cinerea*. Molecules and Cells 24: 232-239.

111. Soulie MC, Perino C, Piffeteau A, Choquer M, Malfatti P, et al. (2006) *Botrytis cinerea* virulence is drastically reduced after disruption of chitin synthase class III gene (*Bcchs3a*). Cellu Microbiol 8: 1310-1321.

112. Stukkens Y, Bultreys A, Grec S, Trombik T, Vanham D, et al. (2005) NpPDR1, a pleiotropic drug resistance-type ATP-binding cassette transporter from *Nicotiana plumbaginifolia*, plays a major role in plant pathogen defense. Plant Physiol 139: 341-352.

113. Tang DZ, Simonich MT, Innes RW (2007) Mutations in LACS2, a long-chain acyl-coenzyme a synthetase, enhance susceptibility to avirulent *Pseudomonas syringae* but confer resistance to *Botrytis cinerea* in Arabidopsis. Plant Physiol 144: 1093-1103.

114. Thoma I, Loeffler C, Sinha AK, Gupta M, Krischke M, et al. (2003) Cyclopentenone isoprostanes induced by reactive oxygen species trigger defense gene activation and phytoalexin accumulation in plants. Plant J 34: 363-375.

115. Thomma BPHJ, Eggermont K, Tierens KFMJ, Broekaert WF (1999) Requirement of functional *ethylene-insensitive 2* gene for efficient resistance of Arabidopsis to infection by *Botrytis cinerea*. Plant Physiol 121: 1093-1101.

116. Thomma B, Nelissen I, Eggermont K, Broekaert WF (1999) Deficiency in phytoalexin production causes enhanced susceptibility of *Arabidopsis thaliana* to the fungus *Alternaria brassicicola*. Plant J 19: 163-171.

117. Thomma BPHJ, Eggermont K, Penninckx IAMA, Mauch-Mani B, Vogelsang R, et al. (1998) Separate jasmonate-dependent and salicylate-dependent defense-response pathways in Arabidopsis are essential for resistance to distinct microbial pathogens. Proc Natl Acad Sci USA 95: 15107–15111.

118. Thuerig B, Felix G, Binder A, Boller T, Tamm L (2005) An extract of *Penicillium chrysogenum* elicits early defense-related responses and induces resistance in *Arabidopsis thaliana* independently of known signalling pathways. Physiol Mol Plant Pathol 67: 180-193.

119. Tierens KFMJ, Thomma BPHJ, Bari RP, Garmier M, Eggermont K, et al. (2002) Esa1, an Arabidopsis mutant with enhanced susceptibility to a range of necrotrophic fungal pathogens, shows a distorted induction of defense responses by reactive oxygen generating compounds. Plant J 29: 131-140.

120. Trotel-Aziz P, Couderchet M, Vernet G, Aziz A (2006) Chitosan stimulates defense reactions in grapevine leaves and inhibits development of *Botrytis cinerea*. Eur J Plant Pathol 114: 405-413.

121. Unger C, Kleta S, Jandl G, von Tiedemann A (2005) Suppression of the defence-related oxidative burst in bean leaf tissue and bean suspension cells by the necrotrophic pathogen *Botrytis cinerea*. J Phytopathol 153: 15-26.

122. Urena AG, Orea JM, Montero C, Jimenez JB, Gonzalez JL, et al. (2003) Improving postharvest resistance in fruits by external application of trans-resveratrol. J Agric Food Chem 51: 82-89.

123. Veronese P, Chen X, Bluhm B, Salmeron J, Dietrich R, et al. (2004) The BOS loci of Arabidopsis are required for resistance to *Botrytis cinerea* infection. Plant J 40: 558-574.

124. Veronese P, Nakagami H, Bluhm B, AbuQamar S, Chen X, et al. (2006) The membrane-anchored BOTRYTIS-INDUCED KINASE1 plays distinct roles in Arabidopsis resistance to necrotrophic and biotrophic pathogens. Plant Cell 18: 257-273.

125. Walley JW, Coughlan S, Hudson ME, Covington MF, Kaspi R, et al. (2007) Mechanical stress induces biotic and abiotic stress responses via a novel cis-element. PLoS Genet 3: 1800-1812.

126. Xing DH, Chen ZX (2006) Effects of mutations and constitutive overexpression of EDS1 and PAD4 on plant resistance to different types of microbial pathogens. Plant Sci 171: 251-262.

127. Yu T, Zheng XD (2007) An integrated strategy to control postharvest blue and grey mould rots of apple fruit by combining biocontrol yeast with gibberellic acid. Int J Food Sci Technol 42: 977-984.

128. Zheng ZY, Abu Qamar S, Chen ZX, Mengiste T (2006) Arabidopsis WRKY33 transcription factor is required for resistance to necrotrophic fungal pathogens. Plant J 48: 592-605.

129. Zimand G, Elad Y, Chet I (1996) Effect of *Trichoderma harzianum* on *Botrytis cinerea* pathogenicity. Phytopathology 86: 1255-1260.

130. Zimmerli L, Metraux JP, Mauch-Mani B (2001) beta-aminobutyric acid-induced protection of Arabidopsis against the necrotrophic fungus *Botrytis cinerea*. Plant Physiol 126: 517-523.

**Publications reporting data on plant—*A. brassicicola* interaction surveyed for isolate information:**

1. Brader G, Djamei A, Teige M, Palva ET, Hirt H (2007) The MAP kinase kinase MKK2 affects disease resistance in Arabidopsis. Mol Plant-Microbe Interact 20: 589-596.

2. Brader G, Mikkelsen MD, Halkier BA, Palva ET (2006) Altering glucosinolate profiles modulates disease resistance in plants. Plant J 46: 758-767.

3. Brodersen P, Petersen M, Nielsen HB, Zhu SJ, Newman MA, et al. (2006) Arabidopsis MAP kinase 4 regulates salicylic acid- and jasmonic acid/ethylene-dependent responses via EDS1 and PAD4. Plant J 47: 532-546.

4. Brouwer M, Lievens B, Van Hemelrijck W, Van den Ackerveken G, Cammue BPA, et al. (2003) Quantification of disease progression of several microbial pathogens on *Arabidopsis thaliana* using real-time fluorescence PCR. FEMS Microbiol Lett 228: 241-248.

5. Chevalier M, Parisi L, Gueye B, Campion C, Simoneau P, et al. (2008) Specific activation of PR-10 pathogenesis-related genes in apple by an incompatible race of *Venturia inaequalis*. Biologia Plantarum 52: 718-722.

6. De Vos M, Van Oosten VR, Van Poecke RMP, Van Pelt JA, Pozo MJ, et al. (2005) Signal signature and transcriptome changes of Arabidopsis during pathogen and insect attack. Mol Plant-Microbe Interact 18: 923-937.

7. De Vos M, Van Zaanen W, Koornneef A, Korzelius JP, Dicke M, et al. (2006) Herbivore-induced resistance against microbial pathogens in Arabidopsis. Plant Physiol 142: 352-363.

8. Flors V, Ton J, van Doorn R, Jakab G, Garcia-Agustin P, et al. (2008) Interplay between JA, SA and ABA signalling during basal and induced resistance against *Pseudomonas syringae* and *Alternaria brassicicola*. Plant J 54: 81-92.

9. Ghose K, Dey S, Barton H, Loake GJ, Basu D (2008) Differential profiling of selected defence-related genes induced on challenge with *Alternaria brassicicola* in resistant white mustard and their comparative expression pattern in susceptible India mustard. Mol Plant Pathol 9: 763-775.

10. Kariola T, Brader G, Li J, Palva ET (2005) Chlorophyllase 1, a damage control enzyme, affects the balance between defense pathways in plants. Plant Cell 17: 282-294.

11. Ko MK, Jeon WB, Kim KS, Lee HH, Seo HH, et al. (2005) A *Colletotrichum gloeosporioides*-induced esterase gene of nonclimacteric pepper (*Capsicum annuum*) fruit during ripening plays a role in resistance against fungal infection. Plant Mol Biol 58: 529-541.

12. Lee SC, Hwang IS, Choi HW, Hwang BK (2008) Involvement of the pepper antimicrobial protein CaAMP1 gene in broad spectrum disease resistance. Plant Physiol 148: 1004-1020.

13. Li CB, Zhao JH, Jiang HL, Wu XY, Sun JQ, et al. (2006) The wound response mutant suppressor of prosystemin-mediated responses6 (spr6) is a weak allele of the tomato homolog of CORONATINE-INSENSITIVE1 (COI1). Plant Cell Physiol 47: 653-663.

14. Liu HZ, Wang XE, Zhang HJ, Yang YY, Ge XC, et al. (2008) A rice serine carboxypeptidase-like gene OsBISCPL1 is involved in regulation of defense responses against biotic and oxidative stress. Gene 420: 57-65.

15. Manners JM, Penninckx I, Vermaere K, Kazan K, Brown RL, et al. (1998) The promoter of the plant defensin gene PDF1.2 from Arabidopsis is systemically activated by fungal pathogens and responds to methyl jasmonate but not to salicylic acid. Plant Mol Biol 38: 1071-1080.

16. McGrath KC, Dombrecht B, Manners JM, Schenk PM, Edgar CI, et al. (2005) Repressor- and activator-type ethylene response factors functioning in jasmonate signaling and disease resistance identified via a genome-wide screen of Arabidopsis transcription factor gene expression. Plant Physiol 139: 949-959.

17. Mengiste T, Chen X, Salmeron J, Dietrich R (2003) The BOTRYTIS SUSCEPTIBLE1 gene encodes an R2R3MYB transcription factor protein that is required for biotic and abiotic stress responses in Arabidopsis. Plant Cell 15: 2551-2565.

18. Miya A, Albert P, Shinya T, Desaki Y, Ichimura K, et al. (2007) CERK1, a LysM receptor kinase, is essential for chitin elicitor signaling in Arabidopsis. Proc Natl Acad Sci USA 104: 19613-19618.

19. Narusaka Y, Narusaka M, Seki M, Ishida J, Nakashima M, et al. (2003) The cDNA Microarray analysis using an Arabidopsis *pad3* mutant reveals the expression profiles and classification of genes induced by *Alternaria brassicicola* attack. Plant Cell Physiol 44: 377-387.

20. Narusaka Y, Narusaka M, Seki M, Umezawa T, Ishida J, et al. (2004) Crosstalk in the responses to abiotic and biotic stresses in Arabidopsis: Analysis of gene expression in cytochrome P450 gene superfamily by cDNA microarray. Plant Mol Biol 55: 327-342.

21. Oh IS, Park AR, Bae MS, Kwon SJ, Kim YS, et al. (2005) Secretome analysis reveals an Arabidopsis lipase involved in defense against *Alternaria brassicicola*. Plant Cell 17: 2832-2847.

22. Penninckx I, Thomma B, Buchala A, Metraux JP, Broekaert WF (1998) Concomitant activation of jasmonate and ethylene response pathways is required for induction of a plant defensin gene in Arabidopsis. Plant Cell 10: 2103-2113.

23. Sarowar S, Lee JY, Ahn ER, Pai HS (2008) A role of hexokinases in plant resistance to oxidative stress and pathogen infection. J Plant Biol 51: 341-346.

24. Schenk PM, Kazan K, Manners JM, Anderson JP, Simpson RS, et al. (2003) Systemic gene expression in Arabidopsis during an incompatible interaction with *Alternaria brassicicola*. Plant Physiol 132: 999-1010.

25. Schenk PM, Kazan K, Rusu AG, Manners JM, Maclean DJ (2005) The SEN1 gene of Arabidopsis is regulated by signals that link plant defence responses and senescence. Plant Physiol Biochem 43: 997-1005.

26. Schenk PM, Kazan K, Wilson I, Anderson JP, Richmond T, et al. (2000) Coordinated plant defense responses in Arabidopsis revealed by microarray analysis. Proc Natl Acad Sci USA 97: 11655-11660.

27. Spoel SH, Johnson JS, Dong X (2007) Regulation of tradeoffs between plant defenses against pathogens with different lifestyles. Proc Natl Acad Sci USA 104: 18842-18847.

28. Staal J, Kaliff M, Dewaele E, Persson M, Dixelius C (2008) RLM3, a TIR domain encoding gene involved in broad-range immunity of Arabidopsis to necrotrophic fungal pathogens. Plant J 55: 188-200.

29. Stintzi A, Weber H, Reymond P, Browse J, Farmer EE (2001) Plant defense in the absence of jasmonic acid: The role of cyclopentenones. Proc Natl Acad Sci USA 98: 12837-12842.

30. Takemoto D, Hardham AR, Jones DA (2005) Differences in cell death induction by Phytophthora elicitins are determined by signal components downstream of MAP kinase kinase in different species of Nicotiana and cultivars of *Brassica rapa* and *Raphanus sativus*. Plant Physiol 138: 1491-1504.

31. Thomma B, Nelissen I, Eggermont K, Broekaert WF (1999) Deficiency in phytoalexin production causes enhanced susceptibility of *Arabidopsis thaliana* to the fungus *Alternaria brassicicola*. Plant J 19: 163-171.

32. Thuerig B, Felix G, Binder A, Boller T, Tamm L (2005) An extract of *Penicillium chrysogenum* elicits early defense-related responses and induces resistance in *Arabidopsis thaliana* independently of known signalling pathways. Physiol Mol Plant Pathol 67: 180-193.

33. Tierens KFMJ, Thomma BPHJ, Bari RP, Garmier M, Eggermont K, et al. (2002) Esa1, an Arabidopsis mutant with enhanced susceptibility to a range of necrotrophic fungal pathogens, shows a distorted induction of defense responses by reactive oxygen generating compounds. Plant J 29: 131-140.

34. Ton J, Mauch-Mani B (2004) beta-amino-butyric acid-induced resistance against necrotrophic pathogens is based on ABA-dependent priming for callose. Plant J 38: 119-130.

35. Trusov Y, Rookes JE, Chakravorty D, Armour D, Schenk PM, et al. (2006) Heterotrimeric G proteins facilitate Arabidopsis resistance to necrotrophic pathogens and are involved in jasmonate signaling. Plant Physiol 140: 210-220.

36. Van der Ent S, Verhagen BWM, Van Doorn R, Bakker D, Verlaan MG, et al. (2008) MYB72 is required in early signaling steps of rhizobacteria-induced systemic resistance in Arabidopsis. Plant Physiol 146: 1293-1304.

37. van Wees SCM, Chang HS, Zhu T, Glazebrook J (2003) Characterization of the early response of Arabidopsis to *Alternaria brassicicola* infection using expression profiling. Plant Physiol 132: 606-617.

38. Veronese P, Chen X, Bluhm B, Salmeron J, Dietrich R, et al. (2004) The BOS loci of Arabidopsis are required for resistance to *Botrytis cinerea* infection. Plant J 40: 558-574.

39. Zheng ZY, Abu Qamar S, Chen ZX, Mengiste T (2006) Arabidopsis WRKY33 transcription factor is required for resistance to necrotrophic fungal pathogens. Plant J 48: 592-605.

40. Zhou CH, Zhang L, Duan J, Miki B, Wu KQ (2005) HISTONE DEACETYLASE19 is involved in jasmonic acid and ethylene signaling of pathogen response in Arabidopsis. Plant Cell 17: 1196-1204.
